# Use of the Capability, Opportunity and Motivation Behaviour model (COM-B) to Understand Interventions to Support Physical Activity Behaviour in People with Stroke: An Overview of Reviews

**DOI:** 10.1177/02692155231224365

**Published:** 2024-01-09

**Authors:** Sarah Paterson, Helen Dawes, Charlotte Winward, Emilia Bartram, Emma Dodds, Jane McKinon, Helen Gaskell, Johnny Collett

**Affiliations:** 1 Centre for Movement, Occupation and Rehabilitation Sciences (MOReS), Faculty of Health and Life Sciences, Oxford Brookes University, Oxford, UK; 2 College of Medicine, Department of Public Health & Sports Sciences, Faculty of Health and Life Sciences, University of Exeter, Medical School Building, College of Medicine and Health, Exeter, UK; 3 Allied Health Professions Research Unit, John Radcliffe Hospital, Oxford, UK; 4 Oxford Centre for Enablement, Nuffield Orthopaedic Centre, Oxford, UK

**Keywords:** Physical activity, stroke, behaviour change, COM-B, overview

## Abstract

**Objective:**

Physical activity in people with stroke remains low despite considerable research. This overview aimed to provide high-level synthesis and aid clinical decision-making. The Capability, Opportunity, Motivation-Behaviour (COM-B) model was used to classify interventions to understand which components improve physical activity behaviour in people with stroke.

**Data Sources:**

CINAHL, Cochrane Database, MEDLINE, PEDro, PsychINFO, SPORTDiscus

**Review Methods:**

A systematic search was conducted (November 2023) to identify reviews of interventions to improve physical activity in people with stroke. Results were screened and assessed for eligibility. Participant characteristics, intervention classification using COM-B, and effect of intervention were extracted. Quality was assessed using AMSTAR2, and Corrected Cover Analysis for study overlap. Narrative synthesis was used to understand components of interventions to improve physical activity behaviour.

**Results:**

1801 references were screened and 29 full-text references assessed for eligibility. Twenty reviews were included. Quality ranged from critically low (*n* = 3) to high (*n* = 10). Study overlap calculated using corrected cover area indicated slight overlap (0.028) and minimal reporting bias.

The majority of participants were mobile with mild stroke and community dwelling. Twenty-three interventions were classified using COM-B. Three of twelve interventions classified to one aspect of the COM-B were effective. Fourteen of sixteen effective interventions combined at least two COM-B elements, ten of these combined capability and motivation.

**Conclusion:**

Interventions including at least two elements of the COM-B are most likely to improve physical activity in mobile stroke survivors. Further research is needed to understand physical activity behaviour in those with moderate to severe stroke.

## Introduction

Exercise has been found to be at least as effective as drug interventions in secondary prevention after stroke,^1^ and physical activity is associated with improved functional recovery, cognition and mood.^2^ Guidelines recommend physical activity promotion should be part of stroke recovery programmes^2^ but studies show that physical activity in people with stroke remains low.^3^

**Figure 1.**
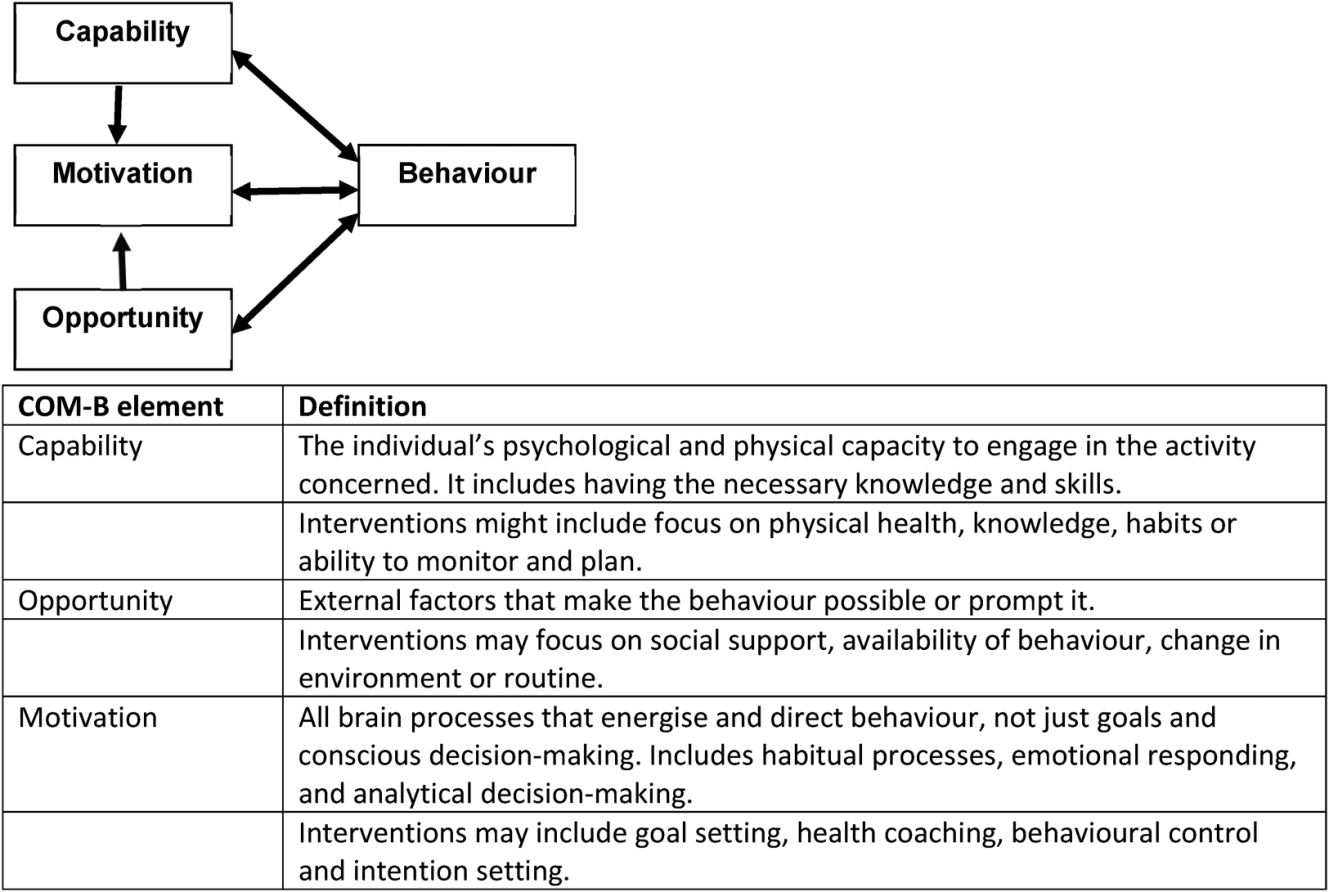
COM-B model of behaviour change.^4,5^

The Capability, Opportunity and Motivation Behaviour model (COM-B) is a theoretical framework to understand and support behaviour change^4^ (Figure 1). In a healthy population, and in people with long-term conditions, the COM-B can predict physical activity, with capability a strong predictor of moderate to vigorous physical activity but some uncertainty about the influence of opportunity and motivation.^5–7^ Given the heterogeneity of physical activity interventions after stroke,^8^ the COM-B may provide a useful framework to understand which components are required for physical activity behaviour change.

There are a large number of reviews of interventions for physical activity after stroke. An overview provides the means to understand what high-level components are important in supporting people with stroke to be more physically active, and gain an understanding of the context of current literature and areas requiring further clarity.^9^

The aim of this overview is to synthesise reviews of interventions to improve physical activity following stroke. It will seek to classify intervention content according to the COM-B model and describe the impact of content on outcomes. In addition, it will describe the characteristics of participants recruited to understand how best to focus physical activity interventions in clinical practice and understand gaps in current evidence base.

## Methods

This overview was designed with reference to the Cochrane guidelines for overviews of reviews,^10^ registered on Prospero (CRD42021268313) and reported considering PRIOR guidelines.^11^

Six databases (MEDLINE, CINAHL, Cochrane Database, PsychINFO, SPORTDiscus, PEDro) were searched (January 2022, updated November 2023) using terms for stroke and physical activity, and limited to systematic reviews (supplementary appendix).

Reviews were eligible if they were systematic, included participants with stroke (with data reported separately for stroke if a sub population), interventions were to improve physical activity or reduce sedentary behaviour (not limited to randomised control trials), and outcomes included physical activity or sedentary behaviour. Non-English language reviews were excluded.

Search results were managed using Rayaan^12^ which identified and removed duplicates prior to screening. All titles and abstracts were screened by SP and an additional reviewer (CW, HG, ED, EB or JM) against the eligibility criteria.

A data extraction table was developed and piloted with four reviews to ensure data was extracted as expected. The table included items on review characteristics, quality assessment using AMSTAR2^13^ and intervention description to enable classification to the COM-B model.^4^ Classification of interventions to capability, opportunity or motivation was determined using the definitions outlined by Michie et al.^4^ (Figure 1) and were described as using one, two or all three elements of the model. SP and an additional independent reviewer (HG, ED, CW, EB or JM) extracted data using the finalised extraction table from all included reviews.

In both screening and data extraction, where discrepancy occurred, consensus was achieved through discussion and a third reviewer consulted if necessary.

Corrected Cover Area was used to determine reporting bias due to inclusion of individual studies in more than one review.^14^ Corrected cover area was defined as 0–5 (slight), 6–10 (moderate), 11–15 (high overlap) and >15 (very high).

Narrative synthesis described participant characteristics, interventions, outcomes and findings on the efficacy of interventions. COM-B was used to classify outcome according to intervention components (see Figure 1).

## Results

**Figure 2.**
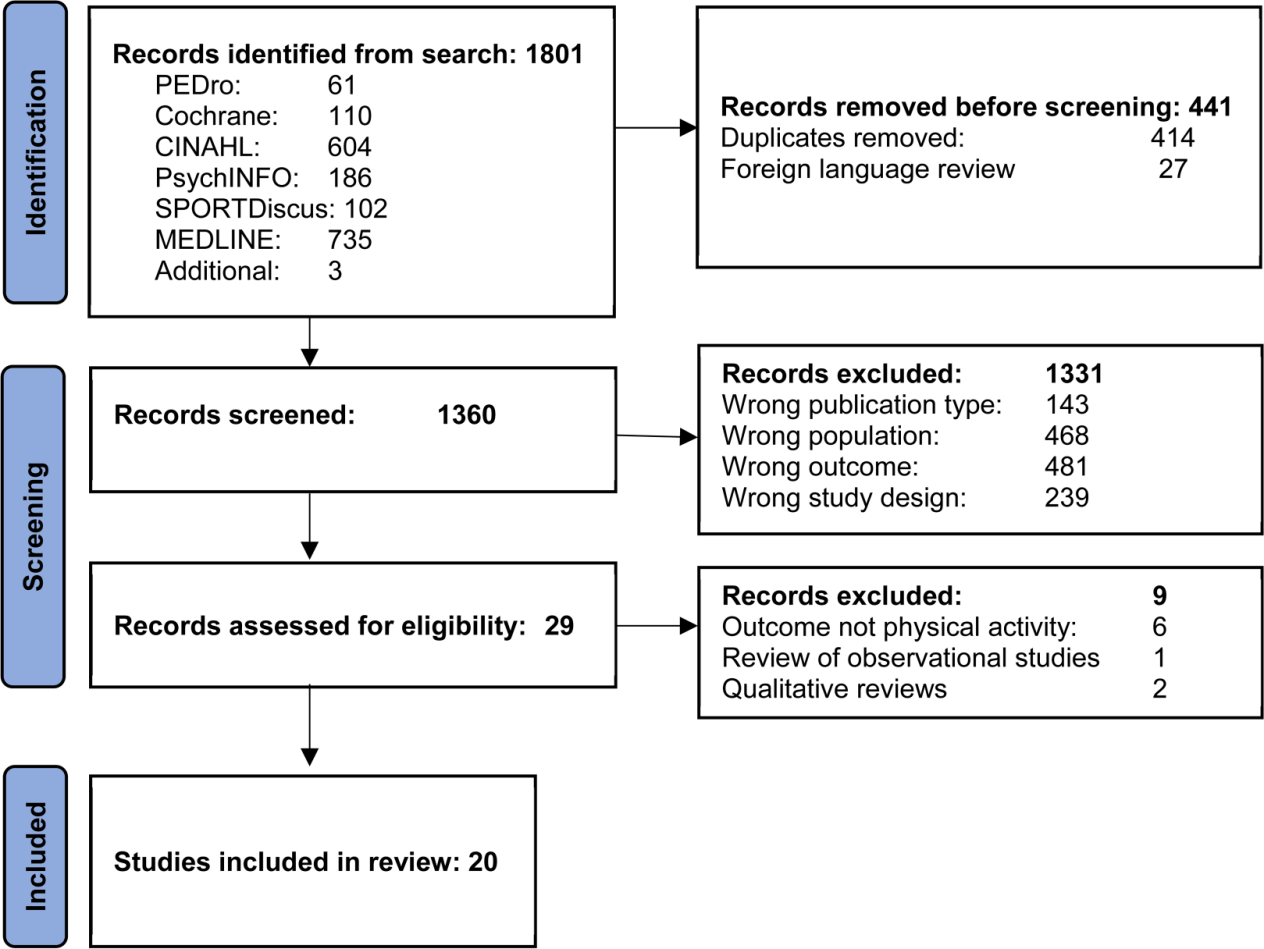
PRISMA flowchart.

The search identified 1801 papers. After removal of duplicates and non-English papers, 1360 titles and abstracts were screened. Eligibility was assessed in 29 full text systematic reviews and 20 included (Figure 2). Of the nine reviews excluded, six did not report physical activity outcomes^15–20^ and three were concerned with factors affecting physical activity not interventions.^21–23^ The included reviews were published between 2011 and 2023, and reported on 252 studies in 260 papers published between 1990 and 2023. In total, the studies included over 22350 participants (two reviews did not report number of participants^24,25^).

Ten reviews were high quality,^26–35^ two moderate,^36,37^ three low^38–40^ and five critically low^24,25,41–43^ (supplemental table 2). Nine included a meta-analysis,^25,28,31,32,35,38,41,42,44^ of these four were high quality,^28,31,32,35^ one moderate quality,^44^ one low quality^38^ and three critically low quality.^25,41,42^

Fifteen reviews included randomised trials only; the remaining five included a mixture of study designs of interventional studies.^24,26,27,40,43^ Fifteen reviews were of interventions to change physical activity, one included physical activity and sedentary behaviour^35^ and one sedentary behaviour.^32^

Although 49 studies were included in more than one systematic review, there was only slight overlap. Corrected cover area was 0.02762. Corrected cover area for reviews of similar intervention was calculated and the intervention type with most overlap was lifestyle interventions (0.07386) indicting slight overlap (Supplemental Tables 3 and 4).

### Review Characteristics (Table 1)

Eleven reviews included adults with first or recurrent stroke, eight included participants with stroke and transient ischaemic attack and one with participants with non-degenerative acquired brain injury.^36^

**Table 1.** Review characteristics (meta-analyses in bold).

Author (year), country of publication	Types of studies, no. studies (no. participants), study dates	Population	Setting	Intervention (PA = **Physical activity) (SB = Sedentary behaviour)**	**Comparison (UC = Usual Care)**	**Outcome measures**
**High Quality Reviews**						
**Barclay et al. (2015), Canada**	**RCT's, 5 (266) 2013–2015**	**Adults (18+) with stroke**	**Community.**	**Walking practice or rehearsal.**	**No intervention, other intervention, UC, placebo**	**Participation; activity level**
Hendrickx et al*.*, (2020), Netherlands	RCTs, 11 (2403) 2009–2018	Adults with stroke or TIA	Not reported	Lifestyle or behavioural intervention	No intervention, other intervention, UC, placebo	PA measure
Lynch et al*.* (2018), Australia	RCTS, randomised cross-over, 4 (245) 2015–2018	Adults (18+) with stroke	Hospital/ community	Activity monitors for PA	No intervention, other intervention, UC, placebo	Steps/ day, time in MVPA, light PA, SB, walking duration, participation.
Moore et al*.* (2018), UK	RCT's, 9 (719) 2003–2016	Adults (18+) with stroke or TIA	Mixed	Interventions targeting free-living PA and /or SB	UC, comparator without PA/ SB component	Changes frequency, duration, intensity of free-living PA or SB at 3 months
**Oliveira et al*.* (2023), Brazil**	**RCT's 28 (1855) 2004–2023**	**Adults (18+) with stroke**	**Hospital/ community**	**Any intervention to improve PA or reduce SB**	**Not reported**	**Objective measures of PA/SB**
**Parrappilly et al*.* (2018), Canada**	**RCT's, 16 (3568) 2005–2017**	**Adults with Stroke or TIA**	**Any**	**Secondary stroke prevention intervention**	**UC, control with fewer activities**	**PA measure**
Powell et al*.* (2016), UK	RCTs, 11 (550) 1994–2015	Adults (18+) with stroke	Not reported	LL wearable technology	UC, Exercise, physical therapy, sham stimulation, conventional gait therapy	Participation; activity level
Sahely et al*.* (2023), UK	No restriction, 24 (823) 1999–2021	Adults (18+) with stroke of any severity.	Not reported	Self-management strategies targeted to improve mobility	Comparator, UC or no comparator	Qualitative or quantitative data related to functional mobility.
Sammut et al*.* (2022), Australia	RCTs or non-randomised trials, 8 (2653)	Adults (18+) with non-disabling stroke or TIA	Community	Interventions to increase time in MVPA	Not reported	Time spent in MVPA, frequency of MVPA, % population meeting guidelines.
**Saunders et al*.* (2021), Scotland**	**RCT's, Cluster RCT, Randomised cross-over, 10 (753) 2004–2019**	**Adults (18+) with stroke**	**Any**	**Any intervention to reduce SB**	**UC, no intervention, sham or adjunct intervention**	** SB/PA levels, participation**
**Moderate quality reviews**						
Jones et al*.* (2015), Australia	RCT/ Quasi-randomized controlled trial, 5 (417) 2007–2013	Adults (18+), non degenerative ABI	Community	Self-management programme including at least one PA component	Not reported	Subjective/objective measure of PA
**Stretton et al*.* (2017), New Zealand**	**Randomised/ Quasi-randomised study design, 9 (1258) 2004–2012**	**Adults (16+) with stroke**	**Not reported**	**Any intervention to change real-world walking**	**UC, no intervention, attention control**	**Measure of real-world walking behaviour.**
**Low quality reviews**						
Aguiar et al*.* (2020), Brazil	RCT's, 18 (1314) 1999–2018	Adults (18+) with stroke	Clinical/ community	Any intervention to improve PA.	Not reported	Subjective/ objective measures of PA
**Lennon et al*.* (2014), Ireland**	**RCTs, 17 (2478) 2000–2011**	**Adults with stroke or TIA**	**Mixed**	**Interventions aimed at changing knowledge, beliefs or behaviours**	**UC, no intervention, sham**	**Measure of PA**
Rintala et al*.* 2022), UK	No restriction, 22 (264)	Adults with mild/ moderate stroke	Not reported	Intervention using a mobile app with physical training component	Any type of control group	Any measure of physical function or physical activity
**Critically low quality reviews**						
**Kringle et al*.* (2020), USA**	Experimental/ quasi-experimental design, 31,1998–2018	Adults with stroke	Non-hospitalised	Any intervention to improve PA	Not reported	Not stated
**Lawrence et al*.* (2011), Scotland**	**RCTs, non-randomised controlled trials, case control, cohort studies, 4 (581) 2005–2009**	**Adults (18+) with stroke or TIA, mixed pop studies with extractable stroke data**	**Not reported**	**Educational/health promotion/ behavioural interventions for recurrent stroke prevention**	**Not reported**	**PA behaviour change**
Morris et al*.* (2014), Scotland	A, 11 (1704) 2006–2012	Adults with stroke	Community-dwelling	Interventions to improve PA, counselling, advice or behaviour-change interventions, with or without exercise	Not reported	Self-report questionnaires, diaries scales or devices of activity or step count
**Pogrebnoy & Dennett (2020), Australia**	**RCTs, 8 (499) 2003–2015**	**Adults with stroke**	**Outpatient community setting**	**Interventions to improve dose, intensity or duration of PA**	**No intervention, UC or exercise programme not meeting guidelines**	**Measure of PA levels**
**Sakakibara et al*.* (2017), Canada**	**RCTs, 14, 2004–2014**	**Adults (18+) with stroke or TIA**	**Not reported**	**Self-management interventions to improve stroke risk factors**	**Not reported**	**Measures of risk factor control including those related to PA**

In five reviews a lack of primary data limited ability to report participant characteristics^30,32,33,38,42^ and in four reviews participant characteristics could not be assertained.^25,30,31,44^ Age ranged from 36.9 to 91 years (reported in 14 reviews), time since stroke was 3.6 days to 25 years (reported in nine reviews).

No reviews specified stroke severity within their inclusion criteria. Eight reviews reported stroke severity in included studies, all of which included only participants with mild to moderate stroke.

Six reviews specifically included studies of community-based interventions,^24,27,28,36,42,43^ seven included community or in-patient studies,^29,31–33,35,38,39^ and seven did not specify setting.^25,26,30,34,40,41,44^

There was a large variety in interventions (Table 2). Most were complex interventions with more than one component. Eleven reviews described behaviour change theory behind interventions.^24–26,32,33,35,36,38,41,43,44^ The COM-B framework was not specifically mentioned. Twenty-three different intervention types were described across the reviews, 11 were classified as capability, 8 as opportunity and 15 as motivation (interventions may incorporate more than one classification). Two reviews could not be classified according to the COM-B due to intervention heterogeneity,^32,39^ and therefore not included in COM-B analysis.

**Table 2.** Impact of COM-B (meta-analyses in bold).

Author, year	AMSTAR rating	Time to follow up	Intervention (BCT = behaviour change technique) SMD (95% confidence interval)	
			Effective	Not shown to be effective
**Capability**				
Moore et al*.,* 2018	High	3–6 months	Supervised strengthening	Aerobic training and education
Pogrebnoy & Dennett, 2020	Critically Low	3–7 months		Aerobic training
**Opportunity**				
Barclay et al*.,* 2015	High	3 weeks – 10 months		Outdoor walking practice: SMD 0.08 (−0.2, 0.35)
**Oliveira et al*.,* 2023**	**High**	**Intervention end – 6** **months**		**Exercise** **SMD −0.03 (−0.55–0.49)**
Sammut et al., 2023	High	Intervention end – 6 months	Exercise on prescription 11.7min/day increase in PA (95% CI 4.07 to 19.33) *P* = 0.031	Exercise on prescription 2.1 min/day increase in PA (95% CI −10.7 to 15.0) *P* = 0.74
**Stretton et al*.* 2017 sub-group**	**Moderate**	**3–12** **months**		**Exercise:** **SMD 0.19 (−0.11,0.49)**
Kringle et al., 2020	Critically Low	Intervention end		Exercise: 6/8 studies no between group difference or within group effect.
Morris et al*.,* 2014	Critically low	3–24 months	Tailored exercise	
**Motivation**				
Lynch et al*.,* 2018	High	Intervention end		Activity monitor feedback
Powell et al*.,* 2016	High	Intervention end to 12 weeks		Activity monitor feedback
Kringle et al., 2020	Critically low	3 months		Behaviour change technique: 1xRCT no between group difference; Cohens *d* (−0.21, 0.33) *P* < 0.05
**Capability – Opportunity**				
Kringle et al., 2020	Critically low	3 months	Exercise - Education Between group effects on PA Cohen's *d* = 0.39, *P* < 0.05.	
**Opportunity – Motivation**				
**Oliveira et al., 2023**	**High**	**Intervention end – 6** **months**	**Exercise-BCT:** **SMD 0.31 (0.23–1.06)**	
**Stretton et al*.* 2017 sub-group**	**Moderate**	**3–12** **months**	**Exercise - BCT:** **SMD 0.27 (0.12, 0.43)**	
Kringle et al., 2020	Critically low	Intervention end – 3 months	Exercise - BCT for PA: 1 study effective	Exercise & BCT: 2 studies ineffective
**Capability – Motivation**				
Moore et al*.*, 2018	High	3–6 months	Self-management programme	
**Parrappilly et al*.;* 2018**	**High**	**Not reported**	**Education - Tailored counselling & Goal-setting:** **Reduction in proportion of physically inactive OR = 0.60 (0.37–0.97)**	
Saheley et al*.,* 2023	High	Not reported	Positive effects of self management interventions (education and motivation) in 6 of 7 studies reporting on PA outcomes	
Hendrickx et al., 2020	Moderate	Not reported	Lifestyle focused on PA: 5/11 showed sig diff; effect size determined in 3 trials SMD 0.29–0.98 Subgroup analysis of specific PA coaching. SMD 0.73 and 0.98	General lifestyle
**Lennon 2014**	**Low**	**3** **months – 2 years**	**Education – counselling focused on PA:** **SMD 0.24 (0.08–0.41)**	**General lifestyle**
Rintala et al*.,* 2022	Low	Not reported	Mobile app based on education and BCT	
Kringle et al., 2020	Critically Low	3 months	Education – BCT: Small within-group effects OR = 2.07, *P* < 0.05	
Lawrence et al., 2011	Critically Low	3–12 months	Education – Goal-setting: Walks/ week OR 0.61, 95%CI .42-.88; Walking participation OR 0.74, 95%CI .45–1.18.	
Morris et al., 2014	Critically Low	3 months – 2 years	Education – Tailored Counselling	
**Sakakibara et al*.,* 2017**	**Critically Low**	**6** **months-2 years**	**Lifestyle interventions for PA:** **SMD 0.08 (-0.08 to 0.24)**	
**Capability – Opportunity – Motivation**				
Jones et al., 2015	Moderate	6 months	Self-management with PA focus	
Kringle et al., 2020	Critically Low	3 months	Education – Exercise - Motivation 1RCT (Cohen's *d* = 0.62, *P* < 0.05	

Primary outcomes varied across reviews. Measures of physical activity were the primary outcome in 13 reviews. These were predominantly accelerometry or validated self-report measures of physical activity. Four reviews focused on secondary prevention and used changes in risk behaviour (including physical activity) as secondary outcomes.^25,31,38,41^ The majority of reviews used wide inclusion of any measure demonstrating change in physical activity (or sedentary behaviour^24,32^). One review specified that outcomes should specifically relate to participation in activity.^28^ Time to follow up ranged between intervention end and two years. Three reviews reported positive results at 2 years following lifestyle interventions focused on physical activity.^25,38,43^

### Effect of COM-B Classification (Table 2)

Three of 12 interventions incorporating only one aspect of the COM-B (capability, opportunity or motivation) were effective.^27,33,43^ These were supervised strengthening (capability), exercise on prescription and tailored exercise (opportunity). Fourteen of 17 interventions were found effective when two aspects of the COM-B were incorporated; ten of these were a combination of capability and motivation,^24–26,31,33,34,38,40,41,43^ three combined opportunity and motivation^24,35,44^ and one combined capability and opportunity.^24^ Two interventions incorporated capability, opportunity and motivation and were effective.^24,36^

*Capability -* Three capability intervention types were synthesised from two reviews.^33,43^ Aerobic training with education,^33^ and aerobic training^43^ were reported to be ineffective. Only a programme of supervised strengthening and conditioning was found to be effective in increasing physical activity time.^33^

*Opportunity -* Six opportunity interventions were synthesised from six reviews,^24,27,28,35,43,44^ these were supervised tailored home exercises,^43^ community walking practice,^28^ and exercise interventions.^24,27,35,44^ The exercise interventions provided opportunity to exercise, rather than specific focus on improving a person's ability (capability) to be more active. Only the supervised tailored home exercises were effective in improving amount of physical activity. Provision of exercise or walking practice alone were ineffective in three reviews. Three reviews^28,35,44^ included meta-analysis and found no pooled significant effect.

*Motivation –* Three motivation interventions were reported from three reviews. Two reviewed activity monitor feedback interventions^29,30^ neither reported a positive effect. The third review included one randomised control trial of a behaviour change intervention with no effect.^24^

*Capability and Motivation -* Twelve intervention types combining capability and motivation were synthesised from ten reviews.^24–26,31,33,34,38,40,41,43^ Ten of these were effective, all consisted of a combination of tailored education and behavioural change techniques focused on physical activity. The two ineffective intervention syntheses^34,38^ were both general lifestyle interventions, not specific to improving physical activity. Three reviews included meta-analysis for capability and motivation interventions, all reported significant effects.^25,31,38^

*Opportunity and Motivation -* Three reviews synthesised interventions consisting of exercise and behavioural change techniques.^24,35,44^ These were found effective where specifically focused on physical activity, but not when interventions were more generalised.^24^ In two reviews, sub-group analysis of interventions combining exercise and behaviour change techniques found that these were more effective (Standard Mean Difference: 0.31, 0.27) than those with exercise alone (−0.03, 0.19).^35,44^

*Capability and Opportunity –* One review^24^ synthesised exercise and education interventions and concluded a positive effect on daily physical activity.

*Capability, Opportunity and Motivation –* Two intervention syntheses incorporating capability, opportunity and motivation were reported as effective. One used a combination of exercise, education and behavioural change techniques^24^ and the other a self-management programme with specific focus on physical activity.^36^

## Discussion

This high-level synthesis suggests evidence best supports interventions combining at least two elements of the COM-B to improve physical activity behaviour after stroke. The majority of evidence was from adults with mild stroke living in the community. Reviews were of varying quality, overlap of studies was slight, indicating minimal reporting bias.

The combination of capability and motivation was most frequently evaluated in reviews. Ten of twelve interventions synthesised from ten reviews found this combination to be effective. This is consistent with the predictive validity of the COM-B that capability and motivation are key drivers for moderate to vigorous physical activity in healthy adults.^5^ Capability was predominantly addressed through education on importance of physical activity and incorporating it into general routine.^24,31,33,38,41,43^ Education was effective in combination with goal setting and social support (motivation and opportunity) to impact physical activity behaviour.^24,38,41^ While opportunity was the most frequently evaluated single aspect intervention, it was least frequently combined. Where opportunity was combined with COM-B aspects, interventions were found to be effective in 6 out of 7 syntheses.

Seven interventions not found to be effective provided a form of training or exercise, using only one aspect of COM-B (capability or opportunity). It may be that these interventions did not provide a meaningful form of activity, therefore limiting sustainable behaviour change.^45^ This may also be reflected where general lifestyle interventions were not found to be effective compared to lifestyle interventions focused on physical activity which were all effective.^34,38^

In the adult population, behaviour change interventions involving motivation, such as goal setting are thought to be more effective than cognitive capability interventions such as education.^46^ Where better understanding is needed due to an ongoing health condition, education as part of a behaviour change intervention can be helpful where activity is known to have a positive outcome on the impact of a condition.^46^

There may be potential to influence behaviours as people gain understanding of long-term health conditions.^47^ A recent qualitative study of stroke survivors and their carers suggested education regarding physical activity including safety and risk, were important factors influencing physical activity early after stroke.^48^ Only nine reviews in this overview reported time since stroke. Education timing may be fundamental when tailoring behaviour change interventions to address barriers such as fear of recurrent stroke,^22,23^ and maximise opportunity of ‘teachable moments’ when the individual may be most receptive to health behaviour advice or risk reducing behaviours.^47^

The nature of overview reviews, combined with heterogeneity of interventions, limits insights that can be gained regarding specific components of interventions. It is possible that interventions containing one element of the COM-B were not found to be effective due to reasons beyond the COM-B model. One high-quality review found supervised strengthening (capability) to be effective with 3–6 month follow up but we were unable to find any consistent differences in synthesis of interventions with only one component which would otherwise explain this finding.

Our results indicate that interventions using behaviour change techniques specifically focused on physical activity alongside education or exercise improve physical activity.^24,25,34,35^ Two reviews in our overview^24,36^ included interventions with all three elements of the COM-B. Whilst this may be optimal, we did not find it essential to improve physical activity behaviour. Given limited resources in healthcare, interventions need to be cost effective. No review in this overview considered cost effectiveness.

Findings of this overview highlight scarcity of evidence to inform how best to support physical activity in those with more severe symptoms following stroke. The impact of impairments such as cognition, fatigue or mood were not reflected in this literature. Stroke survivors’ report fatigue, communication impairment and mood as barriers to participation in physical activity,^49^ and a correlation between fatigue and physical activity has been observed.^50^ While our results support the tailoring of interventions to improve physical activity behaviour, the evidence base is largely limited to those with mild stroke and focuses on impact of physical impairments.

In interpreting results of this overview, it is important to consider the limitation of high-level synthesis. Included reviews had varied inclusion criteria, broad ranging intervention definitions, and a variety of outcome measures. Bias caused by inclusion of the same studies in multiple reviews is a concern in overview reviews.^14^ The included reviews contained 173 different studies with a wide variety of interventions. We had low overlap between reviews of studies, and heterogeneity of the reviews included supported our synthesis according to COM-B components.

Quality of the evidence included varied and was not evaluated in some reviews. Interventions are complex and may not always be most appropriate for investigation using randomised controlled trials^51^ and we did not limit inclusion to randomised controlled trials. Whilst this has implications for risk of bias, 15 out of the 20 reviews in this overview only included randomised controlled trials.

The COM-B has been used to develop rehabilitation interventions after stroke^52,53^ but this is the first time it has been used as a framework for synthesis of intervention components. Whilst other models provide more detailed frameworks to understand interventions, the broad categories of the COM-B enabled synthesis of intervention components related to behaviour change. It is important to consider that components will interact to influence behaviour. Overlap in capability, motivation, opportunity will exist in interventions and when classifying at review level.

This overview demonstrated that at least two elements of the COM-B should be incorporated for a physical activity intervention to be effective and interventions encompassing only one element risk being futile. Our review supports incorporating capability and motivation components when implementing interventions to promote long-term physical activity engagement, and highlighted the relative paucity of evidence to support physical activity behaviour in those with more significant disability after stroke.

**Clinical messages**

- At least two elements of the Capability, Opportunity, Motivation – Behaviour (COM-B) framework should be incorporated into physical activity interventions.
- There is still not sufficient evidence base to understand how best to support physical activity in those with wide-ranging impairment following stroke.

## Supplemental Material

sj-docx-1-cre-10.1177_02692155231224365 - Supplemental material for Use of the Capability, Opportunity and Motivation Behaviour model (COM-B) to Understand Interventions to Support Physical Activity Behaviour in People with Stroke: An Overview of Reviews Supplemental material, sj-docx-1-cre-10.1177_02692155231224365 for Use of the Capability, Opportunity and Motivation Behaviour model (COM-B) to Understand Interventions to Support Physical Activity Behaviour in People with Stroke: An Overview of Reviews by Sarah Paterson, Helen Dawes, Charlotte Winward, Emilia Bartram, Emma Dodds, Jane McKinon, Helen Gaskell and Johnny Collett in Clinical Rehabilitation
