# Supplementary material for: Use of the Capability, Opportunity and Motivation Behaviour model (COM-B) to Understand Interventions to Support Physical Activity Behaviour in People with Stroke: An Overview of Reviews
Source: Clin Rehabil. 2024 Jan 9;38(4):543–57. doi: 10.1177/02692155231224365 (PMC10898199; doi:10.1177/02692155231224365)
Supplement: sj-docx-1-cre-10.1177_02692155231224365 - Supplemental material for Use of the Capability, Opportunity and Motivation Behaviour model (COM-B) to Understand Interventions to Support Physical Activity Behaviour in People with Stroke: An Overview of Reviews [file sj-docx-1-cre-10.1177_02692155231224365.docx]

Supplementary Information

Table 1: Search Strategy

|  | **MEDLINE** | **CINAHL** | **Cochrane Database** | **PsychINFO** | **SPORTDiscus** | **PEDro** |
| --- | --- | --- | --- | --- | --- | --- |
| **Stroke terms** | | | | | | |
| **Key words** | stroke | stroke | stroke | stroke | stroke | stroke |
|  | cerebrovascular accident | cerebrovascular accident | cerebrovascular accident | cerebrovascular accident | cerebrovascular accident |  |
|  | cva | cva | cva | cva | cva |  |
|  | cerebral infarct | cerebral infarct | cerebral infarct | cerebral infarct | cerebral infarct |  |
|  | cerebral h*emorrhage | cerebral haemorrhage | cerebral haemorrhage | cerebral haemorrhage | cerebral haemorrhage |  |
|  | stroke patients | stroke patients | stroke patients | stroke patients | stroke patients |  |
| **Subject headings** | **stroke** | **stroke** | **stroke** | Cerebrovascular accidents | stroke patients |  |
|  | *ischemic stroke* | *cerebral infarction* | *ischemic stroke* |  | stroke treatment |  |
|  | *hemorrhagic stroke* | *hemorrhagic stroke* | *hemorrhagic stroke* |  | stroke |  |
|  | *brain infarction* | *ischemic stroke* | *brain infarction* |  |  |  |
|  | *cerebral infarction* | *embolic stroke* | *cerebral infarction* |  |  |  |
|  | cerebral hemorrhage | **intracranial hemorrhage** | cerebral hemorrhage |  |  |  |
|  |  | *cerebral hemorrhage* | intracranial hemorrhage, hypertensive | |  |  |
|  | intracranial hemorrhage, hypertensive | | stroke rehabilitation |  |  |  |
|  | stroke rehabilitation | stroke patients |  |  |  |  |
|  |  |  |  |  |  |  |
|  | **MEDLINE** | **CINAHL** | **Cochrane Database** | **PsychINFO** | **SPORTDiscus** | **PEDro** |
| **Physical activity terms** | | | | | | |
| **Key words** | "physical activity" | "physical activity" | "physical activity" | "physical activity" | "physical activity" | physical activity |
|  | "physical mobility" | "physical mobility" | "physical mobility" | "physical mobility" | "physical mobility" |  |
|  | "physical function" | "physical function" | "physical function" | "physical function" | "physical function" |  |
|  | "physical exertion" | "physical exertion" | "physical exertion" | "physical exertion" | "physical exertion" |  |
|  | "motor activity" | "motor activity" | "motor activity" | "motor activity" | "motor activity" |  |
|  | "energy expenditure" | "energy expenditure" | "energy expenditure" | "energy expenditure" | "energy expenditure" |  |
|  | sitting | sitting | sitting | sitting | sitting |  |
|  | inactiv* | inactiv* | inactiv* | inactiv* | inactiv* |  |
|  | "sedentary behaviour" | "sedentary behavio*r" | "sedentary behaviour" | "sedentary behaviour" | "sedentary behaviour" |  |
|  | "wearable devices" | "wearable devices" | "wearable devices" | "wearable devices" | "wearable devices" |  |
|  | "fitness tracker*" | "fitness tracker*" | "fitness tracker*" | "fitness tracker*" | "fitness tracker*" |  |
|  | acceleromet* | acceleromet* | acceleromet* | acceleromet* | acceleromet* |  |
| **Subject headings** | walking |  |  | physical activity | physical activity |  |
|  | sedentary behaviour | physical activity | sedentary behaviour |  | pedometers |  |
|  | physical exertion | physical mobility | physical exertion | physical mobility |  |  |
|  | accelerometery | rising |  |  |  |  |
|  | fitness trackers | sitting | fitness trackers |  |  |  |
|  |  | accelerometers |  |  |  |  |
|  |  | fitness trackers |  |  |  |  |
|  |  | walking |  |  |  |  |
|  |  |  |  |  |  |  |
| **Review Terms** | | | | | | |
|  | **MEDLINE** | **CINAHL** | **Cochrane Database** | **PsychINFO** | **SPORTDiscus** | **PEDro** |
| **Key words** | systematic review | systematic review | systematic review | systematic review | systematic review | systematic review |
|  | metaanalysis | metaanalysis | metaanalysis | metaanalysis | metaanalysis |  |
|  | meta-analysis | meta-analysis | meta-analysis | meta-analysis | meta-analysis |  |
|  | "meta analysis" | "meta analysis" | "meta analysis" | "meta analysis" | "meta analysis" |  |
| **Subject headings** | systematic reviews | systematic review | systematic review | systematic review |  |  |
|  | network meta-analysis | meta analysis | network meta-analysis | meta analysis |  |  |
|  | meta analysis | cochrane library | meta analysis |  |  |  |

Table 2: Quality assessment of reviews using AMSTAR2

| Review | 1. | 2. | 3. | 4. | 5. | 6. | 7. | 8. | 9. | 10. | 11. | 12. | 13. | 14. | 15. | 16. | Confidence rating^13^ |
| --- | --- | --- | --- | --- | --- | --- | --- | --- | --- | --- | --- | --- | --- | --- | --- | --- | --- |
| Barclay *et al.,* 2015 | Y | Y | Y | Y | Y | Y | Y | Y | Y | Y | Y | Y | Y | Y | Y | Y | High |
| Hendrickx *et al.,* 2020 | Y | Y | Y | PY | Y | Y | Y | Y | Y | Y | NA | NA | Y | Y | NA | Y | High |
| Lynch *et al.,* 2018 | y | y | y | y | y | y | N | y | y | y | y | y | y | y | y | y | High |
| Moore *et al.* 2018 | Y | Y | Y | Y | Y | Y | Y | Y | PY | N | NA | NA | Y | Y | NA | Y | High |
| Oliveira *et al.,* 2023 | Y | Y | Y | Y | Y | Y | Y | Y | Y | N | Y | Y | Y | Y | N | N | High |
| Parrappilly *et al.,* 2018 | Y | Y | Y | Y | Y | Y | PY | Y | Y | Y | Y | Y | Y | Y | Y | Y | High |
| Powell *et al*., 2016 | Y | Y | Y | Y | Y | Y | Y | Y | Y | N | NA | NA | Y | Y | NA | Y | High |
| Sahely *et al.,* 2023 | Y | Y | Y | Y | Y | Y | N | Y | Y | Y | NA | NA | Y | Y | NA | Y | High |
| Sammut *et al.,* 2023 | Y | Y | Y | Y | Y | Y | N | Y | Y | N | NA | NA | N | Y | NA | Y | High |
| Saunders *et al*., 2021 | Y | Y | Y | Y | Y | Y | Y | Y | Y | N | Y | Y | Y | Y | Y | Y | High |
| Jones *et al.,* 2015 | Y | Y | N | PY | Y | Y | PY | PY | Y | N | NA | NA | Y | Y | NA | Y | Moderate |
| Stretton *et al.,* 2017 | Y | N | Y | Y | Y | Y | Y | Y | Y | Y | Y | Y | Y | Y | PY | Y | Moderate |
| Aguaiar *et al.*, 2020 | Y | Y | Y | Y | Y | Y | N | Y | N | Y | NA | NA | N | N | NA | Y | Low |
| Lennon *et al.,* 2014 | y | PY | N | PY | N | Y | PY | PY | y | N | y | y | Y | y | y | Y | Low |
| Rintala *et al.,* 2022 | Y | N | Y | Y | N | N | N | Y | PY | Y | NA | NA | Y | Y | NA | N | Low |
| Kringle *et al*., 2020 | Y | N | N | PY | Y | Y | y | Y | Y | N | NA | NA | Y | N | NA | Y | Critically low |
| Lawrence *et al.,* 2011 | Y | N | N | Y | N | N | Y | Y | Y | N | Y | Y | Y | Y | N | N | Critically low |
| Sakakibara *et al.,* 2017 | Y | N | Y | Y | Y | Y | Y | PY | N | Y | Y | N | N | N | N | Y | Critically low |
| Pogrebnoy & Dennett, 2020 | Y | Y | N | Y | Y | Y | N | Y | Y | N | n | Y | Y | N | N | N | Critically low |
| Morris *et al,*2014 | Y | N | N | Y | Y | Y | No | Y | Y | N | NA | NA | N | Y | NA | N | Critically low |

Table 3: Studies included in systematic reviews (describing where studies may be used in more than one review)

|  | Aguaiar et al. 2020 | Kringle *et al*., 2020 | Moore et al 2018 | Morris *et al,*2014 | Saunders, *et al.,* 2021 | Barclay *et al.*  2015 | Oliverira *et al.* 2023 | Stretton *et al.,* 2017 | Pogrebnoy & Dennett, 2020 | Lynch *et al.* 2018 | Powell *et al.,* 2016 | Rintala et al., 2022 | Hendrickx et al; 2020 | Lawrence *et al;* 2011 | Lennon *et al,* 2014 | Parrappilly *et al;* 2018 | Sammut *et al.,* 2022 | Jones et al 2015 | Sahely *et al*, 2023 | Sakakibara *et al.,* 2017 | TOTAL |
| --- | --- | --- | --- | --- | --- | --- | --- | --- | --- | --- | --- | --- | --- | --- | --- | --- | --- | --- | --- | --- | --- |
| **No. Studies** | **18** | **31** | **9** | **11** | **8** | **5** | **28** | **10** | **8** | **4** | **11** | **11** | **11** | **3** | **17** | **16** | **8** | **5** | **24** | **14** | **252** |
| **No. Papers** | **18** | **31** | **9** | **11** | **12** | **5** | **28** | **10** | **10** | **4** | **11** | **12** | **11** | **4** | **17** | **16** | **8** | **5** | **24** | **14** | **260** |
| Sit *et al.,* 2007 |  | 1 | 1 | 1 |  |  |  |  |  |  |  |  |  | 1 |  | 1 | 1 | 1 |  |  | 7 |
| Mudge *et al.,* 2009 | 1 | 1 | 1 | 1 |  |  | 1 | 1 |  |  |  |  |  |  |  |  |  |  |  |  | 6 |
| Boysen *et al.,* 2009 | 1 | 1 |  | 1 |  |  |  |  |  |  |  |  |  |  | 1 |  |  |  |  | 1 | 5 |
| Dean *et al.,* 2012 |  | 1 |  | 1 |  |  | 1 | 1 |  |  |  |  |  |  |  |  |  |  |  |  | 4 |
| Duncan *et al.,* 2011 |  | 1 |  | 1 |  |  | 1 | 1 |  |  |  |  |  |  |  |  |  |  |  |  | 4 |
| English *et al.,* 2016 | 1 | 1 |  |  | **1** |  | 1 |  |  |  |  |  |  |  |  |  |  |  |  |  | 4 |
| Gillham *et al.,* 2010 |  |  |  | 1 |  |  |  |  |  |  |  |  | 1 |  | 1 |  |  |  |  | 1 | 4 |
| Green *et al.,*2007 |  |  |  | 1 |  |  |  |  |  |  |  |  |  |  | 1 | 1 |  |  |  | 1 | 4 |
| Kono *et al.,*2013 | 1 |  |  |  |  |  | 1 |  |  |  |  |  | 1 |  |  |  | 1 |  |  |  | 4 |
| Olney *et al.,*  2006 |  | 1 | 1 | 1 |  |  |  |  |  |  |  |  |  |  |  |  |  |  | 1 |  | 4 |
| Allen *et al* 2009 |  |  |  |  |  |  |  |  |  |  |  |  | 1 |  | 1 | 1 |  |  |  |  | 3 |
| Askim *et al.,* 2018 |  | 1 |  |  | 1 |  |  |  |  |  |  |  | 1 |  |  |  |  |  |  |  | 3 |
| Damush *et al.,* 2011 |  |  | 1 |  |  |  |  |  |  |  |  |  |  |  |  |  |  | 1 |  | 1 | 3 |
| Dorsche *et al.,* 2015 |  |  |  |  |  |  | 1 |  |  | **1** | 1 |  |  |  |  |  |  |  |  |  | 3 |
| Givon *et al.,* 2016 | 1 | 1 |  |  |  |  | 1 |  |  |  |  |  |  |  |  |  |  |  |  |  | 3 |
| Joubert *et al.,* 2008 |  |  |  |  |  |  |  |  |  |  |  |  | 1 | 1 | 1 |  |  |  |  |  | 3 |
| Kanai *et al.,*  2018 | 1 |  |  |  |  |  | 1 |  |  | **1** |  |  |  |  |  |  |  |  |  |  | 3 |
| Logan et al., 2004 |  | 1 |  |  |  | 1 |  | 1 |  |  |  |  |  |  |  |  |  |  |  |  | 3 |
| Pang *et al.,*  2005 |  | 1 |  |  |  |  |  | 1 | 1 |  |  |  |  |  |  |  |  |  |  |  | 3 |
| Paul *et al.,* 2018 |  | 1 |  |  |  |  |  |  |  |  |  | 1 |  |  |  |  |  |  | 1 |  | 3 |
| Severinsen *et al.,* 2014 | 1 | 1 | 1 |  |  |  |  |  |  |  |  |  |  |  |  |  |  |  |  |  | 3 |
| Adie *et al.,* 2010 |  |  |  |  |  |  |  |  |  |  |  |  | 1 |  |  |  |  |  |  | 1 | 2 |
| Chiong *et al.,* 2013 | 1 |  |  |  |  |  | 1 |  |  |  |  |  |  |  |  |  |  |  |  |  | 2 |
| Danks et al., 2016 |  |  |  |  |  |  | 1 |  |  | **1** |  |  |  |  |  |  |  |  |  |  | 2 |
| Dean *et al.,* 2018 |  | 1 |  |  |  |  |  |  |  |  |  |  |  |  |  |  |  |  | 1 |  | 2 |
| Ellis *et al.,* 2005 |  |  |  |  |  |  |  |  |  |  |  |  |  |  |  | 1 |  |  |  | 1 | 2 |
| Ellis et al., 2005 |  |  |  |  |  |  |  |  |  |  |  |  |  | 1 | 1 |  |  |  |  |  | 2 |
| Ezeugwu *et al.,* 2018 |  | 1 |  |  |  |  |  |  |  |  |  |  |  |  |  |  |  |  | 1 |  | 2 |
| Faulkner *et al.,* 2015 |  | 1 |  |  |  |  |  |  |  |  |  |  | 1 |  |  |  |  |  |  |  | 2 |
| Flemming *et al.,* 2013 |  |  |  |  |  |  |  |  |  |  |  |  | 1 |  |  | 1 |  |  |  |  | 2 |
| Heron *et al.,* 2019 |  |  |  |  | 1 |  |  |  |  |  |  |  |  |  |  |  | 1 |  |  |  | 2 |
| Hornnes *et al.,* 2011 |  |  |  |  |  |  |  |  |  |  |  |  |  |  | 1 | 1 |  |  |  |  | 2 |
| Krawcyk *et al.,* 2019 |  |  |  |  | 1 |  | 1 |  |  |  |  |  |  |  |  |  |  |  |  |  | 2 |
| Langhammer *et al.,* 2007 |  |  |  | 1 |  |  |  |  | 1 |  |  |  |  |  |  |  |  |  |  |  | 2 |
| Lee *et al.,* 2008 |  |  |  |  |  |  |  |  | 1 |  |  |  |  |  | 1 |  |  |  |  |  | 2 |
| Mansfield *et al.,* 2015 |  |  |  |  |  |  |  | 1 |  | **1** |  |  |  |  |  |  |  |  |  |  | 2 |
| Mansfield *et al.,* 2015 |  |  |  |  |  |  | 1 |  |  |  | 1 |  |  |  |  |  |  |  |  |  | 2 |
| McManus *et al.,* 2009 |  |  |  |  |  |  |  |  |  |  |  |  |  | 1 | 1 |  |  |  |  |  | 2 |
| Moren *et al.,* 2016 |  |  | 1 |  |  |  |  |  |  |  |  |  |  |  |  |  | 1 |  |  |  | 2 |
| Nir *et al.,*2004 |  |  |  |  |  |  |  |  |  |  |  |  |  |  |  | 1 |  |  |  | 1 | 2 |
| Preston *et al.,* 2017 |  | 1 |  |  |  |  |  |  |  |  |  |  |  |  |  |  |  |  | 1 |  | 2 |
| Shaughnessy *et al.,* 2012 | 1 | 1 |  |  |  |  |  |  |  |  |  |  |  |  |  |  |  |  |  |  | 2 |
| Sullivan *et al.,* 2014 |  | 1 |  |  |  |  |  |  |  |  |  |  |  |  |  |  |  |  | 1 |  | 2 |
| Teuschl *et al.,* 2017 | 1 |  |  |  |  |  |  |  |  |  |  |  | 1 |  |  |  |  |  |  |  | 2 |
| Vahlberg *et al.,* 2017 | 1 | 1 |  |  |  |  |  |  |  |  |  |  |  |  |  |  |  |  |  |  | 2 |
| Van der Ploeg *et al.,* 2007 |  |  |  | 1 |  |  |  |  |  |  |  |  |  |  | 1 |  |  |  |  |  | 2 |
| Vanroy *et al.,* 2017 | 1 |  |  |  |  |  | 1 |  |  |  |  |  |  |  |  |  |  |  |  |  | 2 |
| Vloothuis et al, 2019 |  |  |  |  |  |  |  |  |  |  |  | 1 |  |  |  |  |  |  | 1 |  | 2 |
| Wan *et al.,* 2016 | 1 |  |  |  |  |  |  |  |  |  |  |  |  |  |  | 1 |  |  |  |  | 2 |
| Number of papers used with no overlap | 5 | 4 | 2 | 3 | 11 | 0 | 7 | 0 | 3 | 1 | 8 | 7 | 9 | 8 | 7 | 4 | 17 | 5 | 9 | 14 | 124 |

Table 4: Corrected Cover Area calculations to describe study overlap

| **Intervention type** | **Reviews included** | **CCA** | | |
| --- | --- | --- | --- | --- |
| Any | Aguiar, Kringle, Moore, Morris, Saunders | 0.07661 | 0.05263 | 0.02762 |
| Any - focus on community ambulation/ exercise | Barclay, Oliveira, Stretton, | 0.07661 |  |  |
| Exercise guidelines | Pogrebnoy | NA 1 review |  |  |
| Activity monitors | Lynch, Powell | 0.07143 | 0.01351 |  |
| Digital | Rintala | NA 1 review |  |  |
| Lifestyle | Hendrickx, Lawrence, Lennon, Parappilly, Sammut | 0.07386 | 0.03797 |  |
| Self management | Jones, Sahely, Sakakibara | 0.0119 |  |  |

Table 5: Study aims of included reviews

| Author (year), country of publication | Aims/ Research Question |
| --- | --- |
| High quality studies | |
| Barclay *et al* (2015), Canada | Do interventions improve community ambulation for stroke survivors? Does any specific intervention method improve community ambulation more than others? |
| Hendrickx *et al*, (2020), Netherlands | What is the effect of lifestyle interventions on the level of physical activity performed by people with stroke or TIA? |
| Lynch *et al* (2018), Australia | Summarise evidence regarding effectiveness of wearable activity monitors and smartphone apps for increasing PA levels |
| Moore *et al* (2018), UK | Review the study characteristics and the promise of interventions targeting free-living PA and/or sedentary behaviour in adult stroke survivors |
| Oliveira *et al* (2023), Brazil | Determine which interventions increase physical activity and decrease sedentary behaviour based on objective measures of movement behaviour in individuals with stroke. |
| Parrappilly *et al* (2018), Canada | Determine effectiveness of interventions where nurses have primary role on modification of stroke risk factors |
| Powell *et al* (2016), UK | Examine how effective external wearable devices to improve function of the lower limb in adult stroke survivors |
| Sahely *et al* (2023), UK | Provide an evidence synthesis of the self management-based interventions to improve mobility and the relevant outcomes studied within stroke rehabilitation. What are the effects and outcomes of self-management interventions related to the rehabilitation of mobility post stroke? |
| Sammut *et al* (2023), | Determine interventions or strategies that increase time in MVPA. |
| Saunders *et al* (2021), Scotland | Determine whether interventions designed to reduce sedentary behaviour after stroke can reduce risk of death or secondary vascular events, modify CV risk and decrease sedentary behaviour |
| Moderate quality studies | |
| Jones *et al* (2015), Australia | How effective are self-management programmes in improving PA? Which features of a self-management programme are associated with the best clinical outcomes and client satisfaction? |
| Stretton *et al*  (2017), New Zealand | Determine the effectiveness of current interventions to improve real-world walking for people with stroke and whether benefits are sustained |
| Low quality reviews | |
| Aguiar *et al* (2020), Brazil | Identify interventions to increase post-stroke PA. |
| Lennon *et al* (2014), Ireland | Examine the impact of lifestyle interventions on secondary prevention in stroke and TIA |
| Rintala *et al* (2022), UK | Evaluate the effectiveness of mobile health applications containing a physical training component on physical function and PA in stroke rehabilitation |
| Critically low quality reviews | |
| Kringle *et al* (2020), USA | Describe non-pharmacological intervention approaches to reduce SB or promote daily PA. 2. Identify effects of non-pharmacological interventions SB or level of daily PA among people with chronic stroke. |
| Lawrence *et al* (2011), Scotland | Establish whether lifestyle interventions designed to help prevent recurrent stoke are effective in positive changes to lifestyle risk factors. |
| Morris *et al* (2014), Scotland | Investigate the effects of interventions to promote long term participation in PA on measures of frequency, duration, intensity of PA at 3 months or longer post stroke in community dwelling stroke survivors |
| Pogrebnoy et al (2020), Australia | Determine the effectiveness of combined aerobic and resistance training exercise programs, prescribed according to guidelines, for improving mobility and physical activity levels of people with stroke. |
| Sakakibara *et al (*2017), Canada | Describe self-management interventions used to improve risk factor control in stroke and quantitatively assess effects on: 1) overall risk factor control from lifestyle behaviour, and medical risk factors and 2) individual risk factors |

Table 6: Outcomes used in included reviews

| **Review** | **Included outcome measure criteria for physical activity.** | **Outcomes for physical activity used in studies** |
| --- | --- | --- |
| Aguaiar *et al.* 2020 | Any measure of increase in physical activity.  Walking/ exercise capacity, gait patterns, ability to perform ADL or sedentary time excluded. | 8 studies used physical activity scales; 6 used accelerometers inch no. steps and time in low, mod and high intensity activity, arm activity ratio, energy expenditure. |
| Barclay *et al.* 2015 | Primary outcome to focus on participation. Secondary outcomes include activity level outcomes such as gait speed, walking endurance and ability to walk in different environments. | Primary outcomes: Nottingham leisure questionnaire; subjective index of physical and social outcome. Secondary: community walk test and walking ability questionnaire, gait speed, 6-minute walk test, self-efficacy. |
| Hendrickx *et al.* 2020 | At least one measure of PA (any form of light PA and or moderate to vigorous PA). | Accelerometer (steps per day, time in MVPA), self-reported exercise frequency, validated physical activity questionnaire e.g. IPAQ (International Physical Activity Questionnaire). |
| Jones *et al*. 2015 | Measure of physical activity either an activity monitoring device or self-report measure, and/or a study outcome associated with physical activity e.g. physical activity self-efficacy, physical self-concept, stages of change in relation to physical activity. | HPLP-III physical activity subscale, SRAHO Physical activity and exercise domain, PART-O, self reported time spent in aerobic activity SSQOL, CABS-R exercise subscale, SOEQ, physical activity MET minutes/week, general self efficacy scale, participation in walking exercise. |
| Kringle *et al.* 2020 | Primary or secondary outcome measures of sedentary behaviour or level of daily physical activity. | Mix of questionnaire based measures (The CHAMPS Physical Activity Questionnaire for Older Adults, Human Activity Profile, International Physical Activity Questionnaire,35 Multimedia Activity Recall for Children), activity checklists and device based measures (step counters, Actigraph, Sensewear armbands, GENEActiv, ActivPAL) |
| Lawrence *et al.* 2011 | Primary outcomes including measures of change in behaviour relating to physical activity. | 2 studies used deliberate walking for exercise as a PA measure. |
| Lennon *et al.* 2014 | Outcome of interest was secondary prevention including physical activity participation. | PASIPD(physical activity scale for individuals with disability), number participating in sport, sport score (intensity and duration), no. meeting activity guideline, Physical activity disability scale (PADS), change in no. engaging in PA, no. walks, ex frequency, PA scale for elderly (PASE) |
| Lynch *et al.* 2018 | Primary outcomes: steps per day, time in moderate-to-vigorous intensity activity. Secondary outcomes: sedentary time, time spent in light intensity physical activity, walking duration, fatigue, mood, quality of life, community participation & adverse events. | PA outcomes: Steps per day, time in light, mod and vig PA, total time walking, SIS-16, , time spent in 2 min or more walking bouts, number of walking bouts of more than 5 mins, longest bout duration, energy expenditure, functional ambulation category,, stroke self-efficacy score, walking time per day over course of trial. |
| Moore *et al*. 2018 | Report on changes in free-living PA or SB measured in terms of frequency, duration, intensity either objectively or subjectively at least 3 months post intervention. | Accelerometers (2); Subjective measures inch self-management exercise behaviour frequency, Frenchay activities index, walking training frequency and duration measured through questionnaire; physical activity primary outcome (3), secondary outcome (6). |
| Morris *et al.* 2014 | Outcomes included any measure of frequency, duration, or intensity of PA at 3 months or longer, energy expenditure, or functional walking capacity. | Participation in PA, daily step count. |
| Oliveira *et al.* 2023 | Objective measures of physical activity or sedentary behaviour. | Daily step count, time spent in physical activity, time spent in MVPA, time spent in sedentary behaviour, number of bouts of sedentary behaviour, length of bouts of sedentary behaviour. |
| Parrappilly *et al.* 2018 | Primary outcomes of risk factors for stroke including behaviour change in relation to physical activity. | Ratio people physically inactive. |
| Pogrebnoy *et al.* 2020 | Mobility outcome measured objectively e.g. 6MWT, TUG or improve physical activity levels. | Physical activity outcomes: physical activity using Physical Activity Scale for Individuals with Physical Disabilities Related outcomes: habitual walking speed, walking endurance, TUAG, stair climb, sit to stand. |
| Powell et al 2016 | WHO measures of activity or participation. | Measures of PA: average daily walking time, changes in walking duration, daily walking activities, SIS, SSEQ. 19 outcome measures used. Activity and participation measures: FAC, 10MWT, RMI, Nottingham ADL, BI, BBS, 6MWT, SIS, TUG, EFAP, SPPB. |
| Rintala *et al*. 2022 | Any type of outcome measure of physical function or physical activity. | Physical activity outcomes: outdoor walking time, sitting time, number of steps per day and sedentary time. PA measured in 2 studies. |
| Sahely *et al.* 2023 | Qualitative or quantitative data related to functional mobility. In addition to other self-management related outcomes i.e. psychological, social or general well-being measures. | ADL’s, walking capacity, sedentary time, number of steps & time spent stepping. Index of PA using number of steps daily, self-efficacy for physical activity scale. |
| Sammut *et al.* 2022 | Time spent in MVPA; frequency spent engaging in MVPA; % sample population engaging in PA within guideline recommendations; self-reporting of PA; steps per day. | Accelerometry, self-reported physical activity, steps per day, self-reported number of days per week engaging in at least 30 mins of exercise, proportion of participants engaging in at least 30 mins of exercise. |
| Saunders *et al.* 2021 | Outcomes of sedentary behaviour; amount of sedentary time, obtained with any objective, self-reported and/or proxy measures. Reports of interruption in SB. | Measures of sedentary time included objective measures of sedentary time using accelerometry or self-reported measures (multimedia activity recall for children and adults; physical activity scale, IPAQ). Sitting time accumulated in bouts of more than 30 mins, no sit to stand transitions. |
| Sakakibara *et al.* 2017 | Overall risk factor control including measures of participation in physical activity. | PA (min/week); PASE; US behavioural survey question on minutes of moderate physical activity; weekly number ex sessions; shift from passive to active stage in physical activity; self-report regular exercise. |
| Stretton *et al.*  2017 | Measure of real-world walking behaviour. | Variety of self-report measures e.g. RMI, NEADL and PASID. Pedometer, StepWatch activity monitor. |
